# Supplementary material for: Multiple plant diversity components drive consumer communities across ecosystems
Source: Nat Commun. 2019 Mar 29;10:1460. doi: 10.1038/s41467-019-09448-8 (PMC6440984; doi:10.1038/s41467-019-09448-8)
Supplement: Supplementary file 1 — Supplementary Information [file 41467_2019_9448_MOESM1_ESM.pdf]

SUPPLEMENTARY INFORMATION

Multiple plant diversity components drive consumer communities across ecosystems

Schuldt et al.

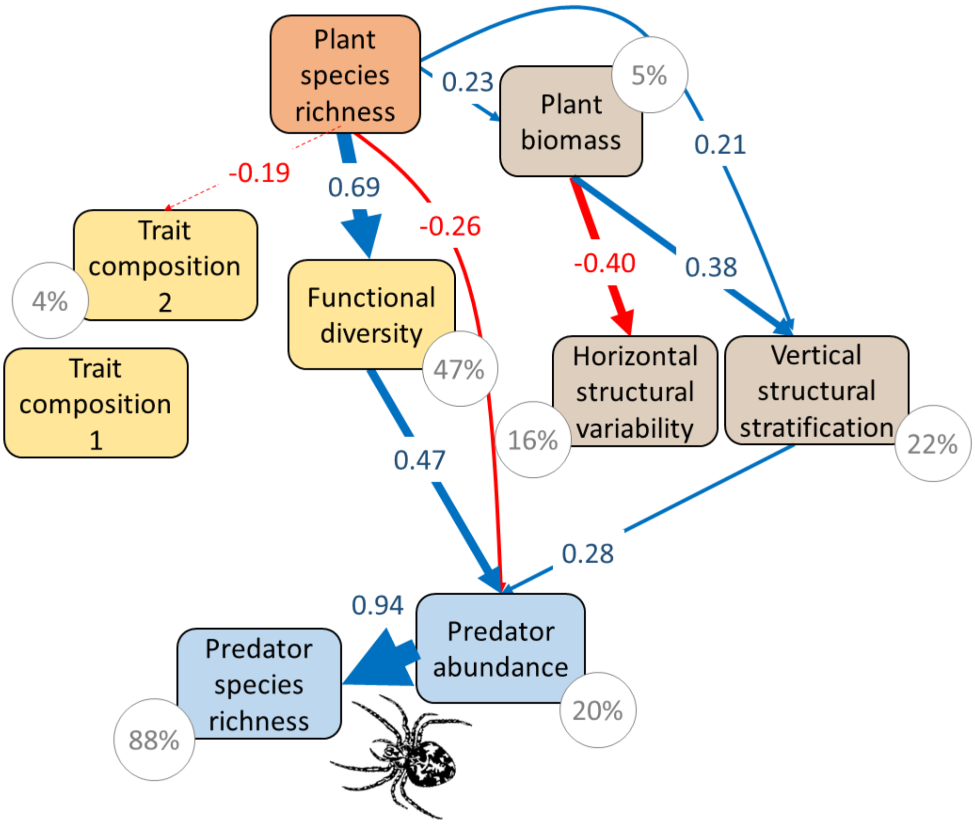

Supplementary Figure 1. Path model for grassland predators (N = 487 individuals) considering only suction sampling data. Direct and indirect effects of species richness (orange), functional trait diversity and composition (yellow), and structural characteristics (brown) of the plant communities on predator abundance and species richness (blue) based on path model results. Trait composition 1 and 2 represent the first two axes of a PCA on community-weighted means of five leaf traits, functional diversity is the mean pairwise dissimilarity (based on Rao's *Q*) of these traits among study plots. Vertical stratification (based on Rao's *Q*) and horizontal variability of plant structure (based on Moran's *I*) represent variability in plant height within the study plots. Positive and negative pathways and their corresponding standardized path coefficients in c) and d) are indicated in blue and red, respectively. Positive and negative pathways and their corresponding standardized path coefficients are indicated in blue and red, respectively. Solid lines show significant relationships ($P \leq 0.05$ based on 1000 bootstrap draws; scaled by their standardized effects), dotted lines show non-significant pathways. Covariances between structural and functional diversity variables shown in Supplementary Figure 4. Percentage values are explained variance of endogenous variables. The animal icon (from www.openclipart.org) is licensed for use in the public domain without copyright (Creative Commons Zero 1.0). Source data are provided as a Source Data file.

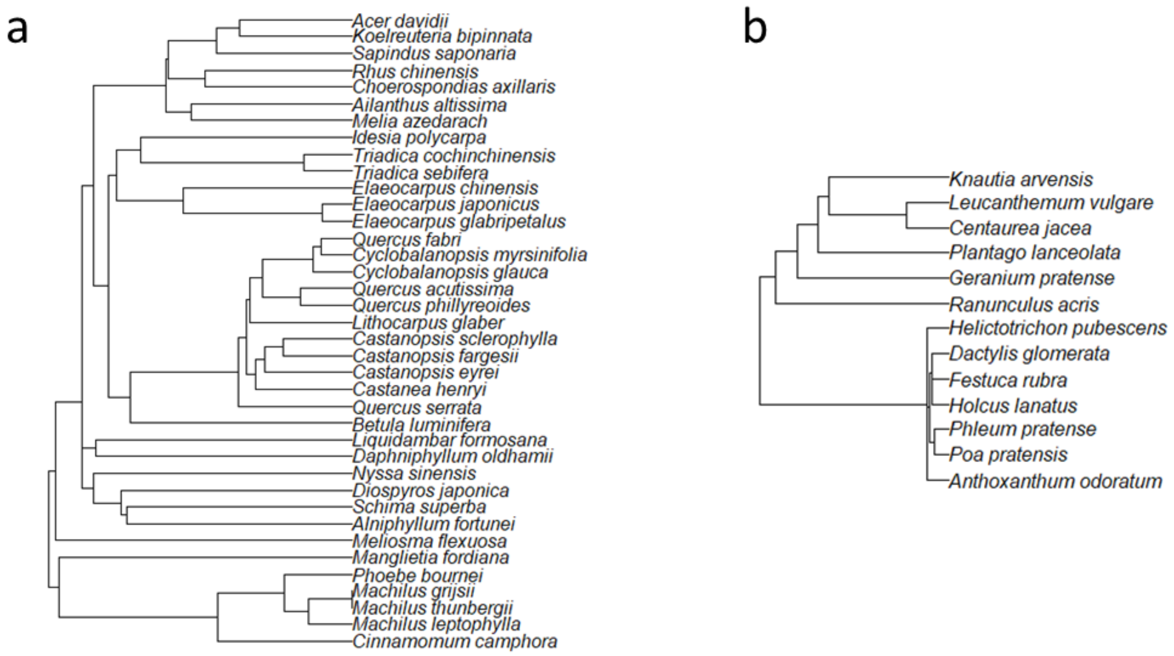

Supplementary Figure 2. Ultrametric phylogenetic trees of the experimental plant communities. Phylogenies, extracted from larger source files, for a) the BEF-China forest experiment and b) the Trait-Based Jena Experiment. Data were extraxted from refs[1,2].

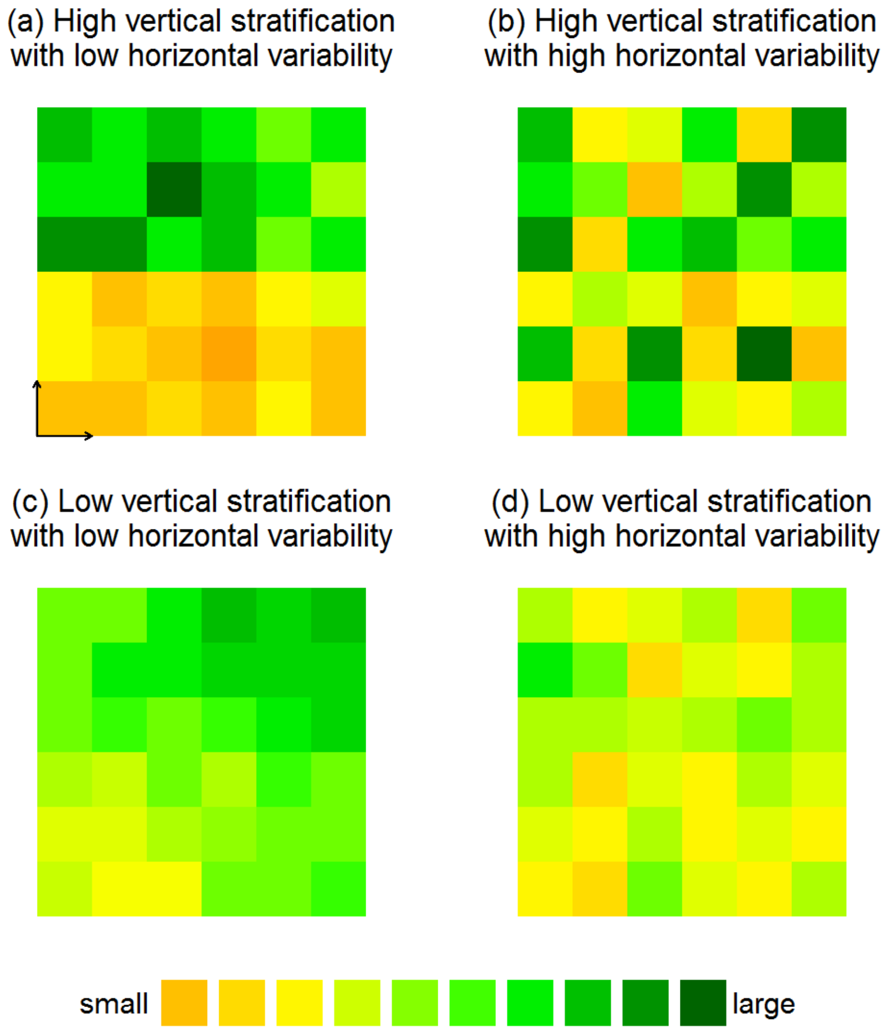

Supplementary Figure 3. Plant structural diversity defined as vertical stratification and horizontal variability. Hypothetical examples depicting the spatial distribution of vertical stratification in plant height (indicated by different colors) and its spatial distribution within study plots in terms of horizontal variability in plant height. Large differences in height between the smallest and the tallest plants results in high vertical stratification (a, b), small differences in low stratification (c, d). This vertical stratification can be spatially clustered within the study plot (a, c) or randomly dispersed across the plot (b, d). High vertical stratification and spatial dispersion were considered to promote structural diversity for arthropod consumers, because the resulting small-scale variability in plant structure is expected to support a higher diversity of habitats and environmental conditions (such as variability in microclimate).

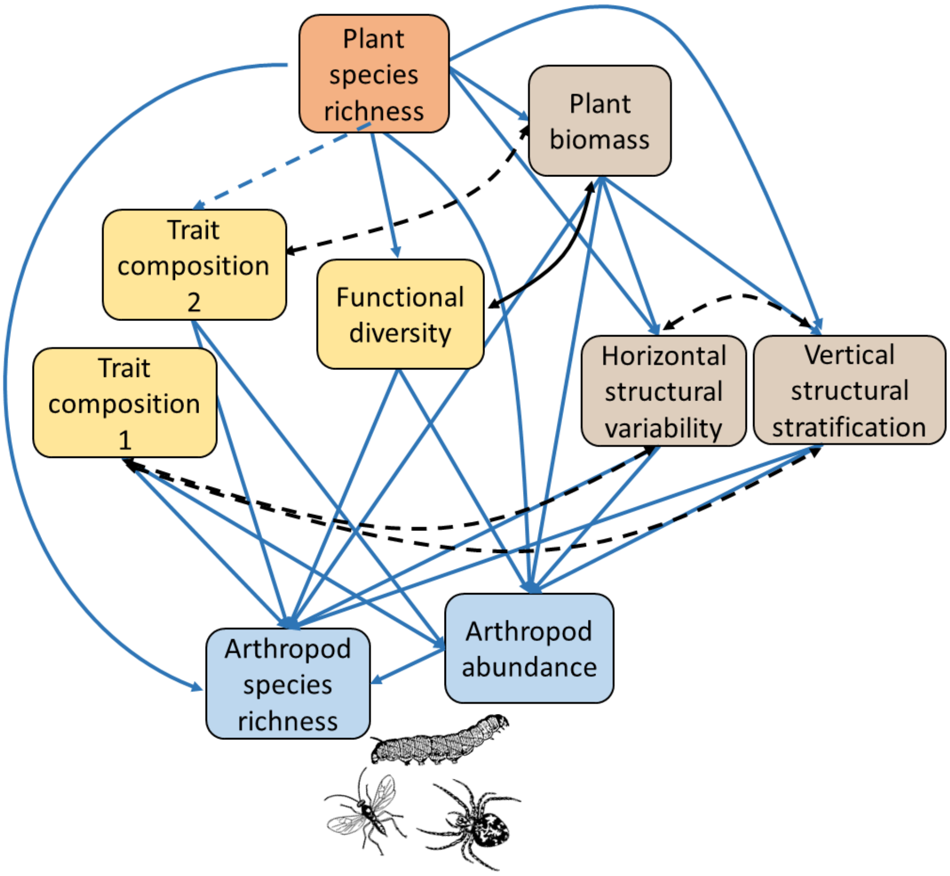

Supplementary Figure 4. **Initial model structure of the path models.** Structure based on theoretical expectations and correlations among plant-based variables: species richness (orange), functional trait diversity and composition (yellow), and structural characteristics (brown). Blue arrows indicate expected causal relationships. Trait composition 1 and 2 represent the first two axes of a PCA on community-weighted means of five leaf traits, functional diversity is the mean pairwise dissimilarity (based on Rao's *Q*) of these traits among study plots. Vertical stratification (based on Rao's *Q*) and horizontal variability of plant structure (based on Moran's *I*) represent variability in plant height (grassland) or the first two axes of a PCA on the variability of tree height and crown projection area (forest) within the study plots. Solid lines are paths fitted in both study systems, broken lines indicate additional paths fitted in the grassland experiment. Black lines are covariances retained in the path models (solid: forest, broken: grassland). The animal icons (from www.openclipart.org) are licensed for use in the public domain without copyright (Creative Commons Zero 1.0).

Supplementary Table 1. Correlations between species richness and abundance across trophic levels.

BEF-China experiment in forests

	Herbivore SR	Predator SR	Parasitoid SR	Herbivore Ind.	Predator Ind.	Parasitoid Ind.
Herbivore SR		0.24	0.04	**0.59**	-0.02	-0.08
Predator SR	0.108		0.03	-0.07	**0.79**	0.03
Parasitoid SR	0.801	0.818		-0.09	0.03	**0.83**
Herbivore Ind.	**< 0.001**	0.622	0.567		-0.12	-0.20
Predator Ind.	0.902	**< 0.001**	0.843	0.412		0.10
Parasitoid Ind.	0.613	0.824	**< 0.001**	0.184	0.525	

Jena Experiment in grasslands

	Herbivore SR	Predator SR	Parasitoid SR	Herbivore Ind.	Predator Ind.	Parasitoid Ind.
Herbivore SR		**0.25**	**0.48**	**0.62**	**0.32**	**0.48**
Predator SR	**0.016**		0.15	0.06	**0.69**	0.17
Parasitoid SR	**< 0.001**	0.166		**0.40**	0.11	**0.88**
Herbivore Ind.	**< 0.001**	0.576	**< 0.001**		**0.24**	0.36
Predator Ind.	**0.002**	**< 0.001**	0.306	**0.022**		0.16
Parasitoid Ind.	**< 0.001**	0.108	**< 0.001**	**< 0.001**	0.138	

Pearson's *r* (upper right part of table) and *P*-values (lower left part), with significant ($P \leq 0.05$) correlations indicated in bold. Correlations based on log-transformed data (and, in the case of the BEF-China experiment in forest, corrected for number of trees sampled; see Methods).

Supplementary Table 2. Path model output for biodiversity effects on *overall arthropod* species richness in the BEF-China *forest experiment*.

**Model**	
AICc initial model	2789.5
AICc minimal model	587.8
Number of observations	46
Chi-square	6.96
P (Chi-square)	0.729
P (Bollen-Stine Bootstrap)	0.789
Degrees of freedom	10
RMSEA	0
95% CI (RMSEA)	0 - 0.118
P (RMSEA)	0.796

Regressions

Respone ~ Predictor	Estimate	SE	*z*	*P* (bootstr.)	Stand. Est.
**Functional diversity ~**					
Tree species richness	0.353	0.046	7.7	< 0.001	0.69
**Tree biomass ~**					
Tree species richness	0.737	0.213	3.5	< 0.001	0.47
**Vertical structural stratification ~**					
Tree biomass	0.647	0.120	5.4	< 0.001	0.71
**Horizontal structural variation ~**					
Tree species richness	-0.440	0.165	-2.7	0.008	-0.34
Tree biomass	0.416	0.117	3.6	< 0.001	0.50
**Arthropod abundance ~**					
Functional diversity	0.264	0.077	3.4	0.001	0.38
Vertical structural stratification	0.073	0.034	2.1	0.034	0.29
**Arthropod species richness ~**					
Tree species richness	0.110	0.026	4.3	< 0.001	0.46
Horizontal structural variation	-0.041	0.019	-2.1	0.032	-0.22
Arthropod abundance	0.250	0.101	2.5	0.014	0.37

Variances

Variable	Estimate	SE	*z*	*P* (bootstr.)	Stand. Est.
Tree species richness	0.835	0.174	4.8	< 0.001	1.00
Functional diversity	0.117	0.028	4.2	< 0.001	0.53
Tree biomass	1.609	0.287	5.6	< 0.001	0.78
Vertical structural stratification	0.834	0.224	3.7	< 0.001	0.49
Horizontal structural variation	1.108	0.185	6.0	< 0.001	0.79
Arthropod abundance	0.080	0.021	3.8	< 0.001	0.75
Arthropod species richness	0.021	0.005	4.2	< 0.001	0.45

Covariances

Variables	Estimate	SE	*z*	*P* (bootstr.)	Stand. Est.
**Functional diversity ~~**					
Tree biomass	-0.14	0.06	-2.5	0.011	-0.32

R^2

Variable	Estimate
Functional diversity	0.47
Tree biomass	0.22
Vertical structural stratification	0.51
Horizontal structural variation	0.21
Arthropod abundance	0.25
Arthropod species richness	0.55

Non-significant (*P* > 0.05 based on 1,000 bootstrap draws) predictors are printed in italics. RMSEA = Root mean square error of approximation.

Supplementary Table 3. Path model output for biodiversity effects on *overall arthropod* species richness in the Jena *grassland experiment*.

**Model**	
AICc initial model	168.9
AICc minimal model	-185.4
Number of observations	92
Chi-square	19.9
P (Chi-square)	0.134
P (Bollen-Stine Bootstrap)	0.185
Degrees of freedom	14
RMSEA	0.067
95% CI (RMSEA)	0 - 0.130
P (RMSEA)	0.304

Regressions

Respone ~ Predictor	Estimate	SE	*z*	*P* (bootstr.)	Stand. Est.
**Functional diversity ~**					
Plant species richness	0.86	0.07	11.8	< 0.001	0.69
**Plant biomass ~**					
Tree species richness	0.14	0.07	2.0	0.044	0.23
**Vertical structural stratification ~**					
Plant species richness	0.03	0.01	2.7	0.008	0.21
Plant biomass	0.09	0.02	4.8	< 0.001	0.38
**Horizontal structural variation ~**					
Plant biomass	-0.04	0.01	-4.4	< 0.001	-0.40
**Arthropod abundance ~**					
Functional diversity	0.08	0.04	2.0	0.047	0.20
Vertical structural stratification	*-0.70*	*0.40*	*-1.7*	*0.083*	*-0.21*
**Arthropod species richness ~**					
Functional diversity	0.06	0.02	3.0	0.003	0.23
Vertical structural stratification	0.38	0.16	2.3	0.022	0.18
Trait composition 1	-0.02	0.01	-2.7	0.007	-0.20
Arthropod abundance	0.42	0.05	9.5	< 0.001	0.66

Variances

Variable	Estimate	SE	*z*	*P* (bootstr.)	Stand. Est.
Plant species richness	0.24	0.04	6.9	< 0.001	1.00
Functional diversity	0.20	0.03	7.2	< 0.001	0.53
Trait composition 1	2.76	0.39	7.1	< 0.001	1.00
Plant biomass	0.08	0.01	7.0	< 0.001	0.95
Vertical structural stratification	0.00	0.00	8.1	< 0.001	0.78
Horizontal structural variation	0.00	0.00	6.7	< 0.001	0.84
Arthropod abundance	0.05	0.01	5.3	< 0.001	0.93
Arthropod species richness	0.01	0.00	5.9	< 0.001	0.42

Covariances

Variables	Estimate	SE	*z*	*P* (bootstr.)	Stand. Est.
**Vertical structural stratification ~~**					
Horizontal structural variation	0.00	0.00	-4.9	< 0.001	-0.51
Trait composition 1	0.05	0.01	4.8	< 0.001	0.43
**Horizontal structural variation ~~**					
Trait composition 1	-0.02	0.01	-3.8	< 0.001	-0.44

R²

Variable	Estimate
Functional diversity	0.47
Plant biomass	0.05
Vertical structural stratification	0.22
Horizontal structural variation	0.16

Arthropod abundance	0.07
Arthropod species richness	0.58

Non-significant ($P > 0.05$ based on 1,000 bootstrap draws) predictors are printed in italics. RMSEA = Root mean square error of approximation.

Supplementary Table 4. Path model output for biodiversity effects on *herbivore* species richness in the BEF-China *forest experiment*.

**Model**					
AICc initial model	2911.3				
AICc minimal model	706.0				
Number of observations	46				
Chi-square	6.6				
P (Chi-square)	0.828				
P (Bollen-Stine Bootstrap)	0.796				
Degrees of freedom	11				
RMSEA	0				
95% CI (RMSEA)	0 - 0.094				
P (RMSEA)	0.878				
**Regressions**					
Respone ~ Predictor	Estimate	SE	*z*	*P* (bootstr.)	Stand. Est.
**Functional diversity ~**					
Tree species richness	0.35	0.05	7.5	< 0.001	0.69
**Tree biomass ~**					
Tree species richness	0.74	0.20	3.7	< 0.001	0.47
**Vertical structural stratification ~**					
Tree biomass	0.65	0.12	5.2	< 0.001	0.71
**Horizontal structural variation ~**					
Tree species richness	-0.44	0.17	-2.5	0.011	-0.34
Tree biomass	0.42	0.12	3.4	< 0.001	0.50
**Arthropod abundance ~**					
Tree species richness	0.22	0.07	3.2	0.001	0.35
**Arthropod species richness ~**					
Tree species richness	0.19	0.06	3.4	0.001	0.39
Horizontal structural variation	-0.09	0.04	-2.3	0.021	-0.26
Arthropod abundance	0.31	0.13	2.4	0.018	0.42
**Variances**					
Variable	Estimate	SE	*z*	*P* (bootstr.)	Stand. Est.
Tree species richness	0.84	0.17	4.8	< 0.001	1.00
Functional diversity	0.12	0.03	4.0	< 0.001	0.53
Tree biomass	1.61	0.28	5.8	< 0.001	0.78
Vertical structural stratification	0.83	0.23	3.6	< 0.001	0.49
Horizontal structural variation	1.11	0.18	6.3	< 0.001	0.79
Arthropod abundance	0.30	0.08	3.7	< 0.001	0.88
Arthropod species richness	0.08	0.02	4.7	< 0.001	0.46
**Covariances**					
Variables	Estimate	SE	*z*	*P* (bootstr.)	Stand. Est.
**Functional diversity ~~**					
Tree biomass	-0.14	0.06	-2.4	0.017	-0.32
R^2					
Variable	Estimate				
Functional diversity	0.47				
Tree biomass	0.22				
Vertical structural stratification	0.51				
Horizontal structural variation	0.21				
Arthropod abundance	0.12				
Arthropod species richness	0.54				

Non-significant (*P* > 0.05 based on 1,000 bootstrap draws) predictors are printed in italics.
RMSEA = Root mean square error of approximation.

Supplementary Table 5. Path model output for biodiversity effects on *predator* species richness in the BEF-China *forest experiment*.

**Model**	
AICc initial model	2799.5
AICc minimal model	778.6
Number of observations	46
Chi-square	13.4
P (Chi-square)	0.706
P (Bollen-Stine Bootstrap)	0.723
Degrees of freedom	17
RMSEA	0
95% CI (RMSEA)	0 - 0.104
P (RMSEA)	0.799

Regressions

Respone ~ Predictor	Estimate	SE	*z*	*P* (bootstr.)	Stand. Est.
**Functional diversity ~**					
Tree species richness	0.35	0.05	7.7	< 0.001	0.69
**Tree biomass ~**					
Tree species richness	0.74	0.22	3.4	0.001	0.47
**Vertical structural stratification ~**					
Tree biomass	0.65	0.12	5.4	< 0.001	0.71
**Horizontal structural variation ~**					
Tree species richness	-0.44	0.17	-2.6	0.008	-0.34
Tree biomass	0.42	0.12	3.4	0.001	0.50
**Arthropod abundance ~**					
Tree biomass	0.14	0.06	2.4	0.018	0.49
Trait composition 1	-0.09	0.03	-3.2	0.001	-0.29
Vertical structural stratification	-0.24	0.06	-3.9	< 0.001	-0.74
**Arthropod species richness ~**					
Functional diversity	0.14	0.05	2.9	0.004	0.24
Arthropod abundance	0.52	0.06	8.0	< 0.001	0.79

Variances

Variable	Estimate	SE	*z*	*P* (bootstr.)	Stand. Est.
Tree species richness	0.84	0.18	4.8	< 0.001	1.00
Functional diversity	0.12	0.03	4.0	< 0.001	0.53
Trait composition 1	2.03	0.64	3.2	0.002	1.00
Tree biomass	1.61	0.29	5.6	< 0.001	0.78
Vertical structural stratification	0.83	0.23	3.7	< 0.001	0.49
Horizontal structural variation	1.11	0.18	6.2	< 0.001	0.79
Arthropod abundance	0.11	0.02	5.4	< 0.001	0.64
Arthropod species richness	0.02	0.00	5.7	< 0.001	0.33

Covariances

Variables	Estimate	SE	*z*	*P* (bootstr.)	Stand. Est.
**Functional diversity ~~**					
Tree biomass	-0.14	0.05	-2.6	0.010	-0.32

R^2

Variable	Estimate
Functional diversity	0.47
Tree biomass	0.22
Vertical structural stratification	0.51
Horizontal structural variation	0.21
Arthropod abundance	0.36
Arthropod species richness	0.67

Non-significant (*P* > 0.05 based on 1,000 bootstrap draws) predictors are printed in italics. RMSEA = Root mean square error of approximation.

Supplementary Table 6. Path model output for biodiversity effects on *parasitoid* species richness in the BEF-China *forest experiment*.

**Model**					
AICc initial model	2917.7				
AICc minimal model	714.6				
Number of observations	46				
Chi-square	7.7				
P (Chi-square)	0.809				
P (Bollen-Stine Bootstrap)	0.814				
Degrees of freedom	12				
RMSEA	0				
95% CI (RMSEA)	0 - 0.096				
P (RMSEA)	0.866				
**Regressions**					
Respone ~ Predictor	Estimate	SE	*z*	*P* (bootstr.)	Stand. Est.
**Functional diversity ~**					
Tree species richness	0.35	0.05	7.5	0.000	0.69
**Tree biomass ~**					
Tree species richness	0.74	0.22	3.4	0.001	0.47
**Vertical structural stratification ~**					
Tree biomass	0.65	0.12	5.4	0.000	0.71
**Horizontal structural variation ~**					
Tree species richness	-0.44	0.17	-2.6	0.010	-0.34
Tree biomass	0.42	0.12	3.3	0.001	0.50
**Arthropod abundance ~**					
Vertical structural stratification	0.23	0.07	3.3	0.001	0.40
**Arthropod species richness ~**					
Vertical structural stratification	0.07	0.03	2.2	0.028	0.19
Arthropod abundance	0.51	0.06	8.8	0.000	0.75
**Variances**					
Variable	Estimate	SE	*z*	*P* (bootstr.)	Stand. Est.
Tree species richness	0.84	0.18	4.7	0.000	1.00
Functional diversity	0.12	0.03	4.1	0.000	0.53
Tree biomass	1.61	0.28	5.8	0.000	0.78
Vertical structural stratification	0.83	0.23	3.7	0.000	0.49
Horizontal structural variation	1.11	0.19	5.8	0.000	0.79
Arthropod abundance	0.47	0.11	4.2	0.000	0.84
Arthropod species richness	0.07	0.02	4.5	0.000	0.28
**Covariances**					
Variables	Estimate	SE	*z*	*P* (bootstr.)	Stand. Est.
**Functional diversity ~~**					
Tree biomass	-0.14	0.06	-2.4	0.015	-0.32
R^2					
Variable	Estimate				
Functional diversity	0.47				
Tree biomass	0.22				
Vertical structural stratification	0.51				
Horizontal structural variation	0.21				
Arthropod abundance	0.16				
Arthropod species richness	0.72				

Non-significant (*P* > 0.05 based on 1,000 bootstrap draws) predictors are printed in italics. RMSEA = Root mean square error of approximation.

Supplementary Table 7. Path model output for biodiversity effects on *herbivore* species richness in the Jena *grassland experiment*.

**Model**					
AICc initial model	410.1				
AICc minimal model	338.72				
Number of observations	92				
Chi-square	25.3				
P (Chi-square)	0.19				
P (Bollen-Stine Bootstrap)	0.259				
Degrees of freedom	20				
RMSEA	0.054				
95% CI (RMSEA)	0 - 0.110				
P (RMSEA)	0.424				
**Regressions**					
Respone ~ Predictor	Estimate	SE	*z*	*P* (bootstr.)	Stand. Est.
**Functional diversity ~**					
Plant species richness	0.86	0.07	11.6	< 0.001	0.69
**Trait composition 2 ~**					
Tree species richness	*-0.46*	*0.27*	*-1.7*	*0.095*	*-0.19*
**Plant biomass ~**					
Tree species richness	0.14	0.07	2.0	0.044	0.23
**Vertical structural stratification ~**					
Plant species richness	0.03	0.01	2.6	0.009	0.21
Plant biomass	0.09	0.02	4.5	< 0.001	0.38
**Horizontal structural variation ~**					
Plant biomass	-0.04	0.01	-4.3	< 0.001	-0.40
**Arthropod abundance ~**					
Functional diversity	0.22	0.07	3.0	0.003	0.28
Vertical structural stratification	-2.23	0.70	-3.2	< 0.001	-0.35
**Arthropod species richness ~**					
Vertical structural stratification	1.41	0.35	4.1	< 0.001	0.37
Trait composition 1	-0.05	0.01	-3.7	< 0.001	-0.29
Trait composition 2	0.06	0.02	3.5	0.001	0.24
Arthropod abundance	0.40	0.06	6.1	< 0.001	0.67
**Variances**					
Variable	Estimate	SE	*z*	*P* (bootstr.)	Stand. Est.
Plant species richness	0.24	0.03	7.4	< 0.001	1.00
Functional diversity	0.20	0.03	7.2	< 0.001	0.53
Trait composition 1	2.76	0.39	7.0	< 0.001	1.00
Trait composition 2	1.34	0.25	5.4	< 0.001	0.96
Plant biomass	0.08	0.01	7.0	< 0.001	0.95
Vertical structural stratification	0.00	0.00	8.7	< 0.001	0.78
Horizontal structural variation	0.00	0.00	7.1	< 0.001	0.84
Arthropod abundance	0.18	0.03	5.4	< 0.001	0.84
Arthropod species richness	0.04	0.01	5.5	< 0.001	0.47
**Covariances**					
Variables	Estimate	SE	*z*	*P* (bootstr.)	Stand. Est.
**Vertical structural stratification ~~**					
Horizontal structural variation	0.00	0.00	-5.2	< 0.001	-0.51
Trait composition 1	0.05	0.01	4.7	< 0.001	0.43
**Horizontal structural variation ~~**					
Trait composition 1	-0.02	0.01	-3.9	< 0.001	-0.44
**Trait composition 2 ~~**					
Plant biomass	-0.11	0.04	-2.6	0.009	-0.35
**R^2**					

Variable	Estimate
Functional diversity	0.47
Trait composition 2	0.04
Plant biomass	0.05
Vertical structural stratification	0.22
Horizontal structural variation	0.16
Arthropod abundance	0.16
Arthropod species richness	0.53

Non-significant ($P > 0.05$ based on 1,000 bootstrap draws) predictors are printed in italics. RMSEA = Root mean square error of approximation.

Supplementary Table 8. Path model output for biodiversity effects on *predator* species richness in the Jena *grassland experiment*.

**Model**	
AICc initial model	199.4
AICc minimal model	-212.2
Number of observations	92
Chi-square	14.8
P (Chi-square)	0.463
P (Bollen-Stine Bootstrap)	0.495
Degrees of freedom	15
RMSEA	0
95% CI (RMSEA)	0 - 0.098
P (RMSEA)	0.679

Regressions

Respone ~ Predictor	Estimate	SE	*z*	*P* (bootstr.)	Stand. Est.
**Functional diversity ~**					
Plant species richness	0.86	0.08	11.3	< 0.001	0.69
**Trait composition 2 ~**					
Plant species richness	*-0.46*	*0.27*	*-1.7*	*0.085*	*-0.19*
**Plant biomass ~**					
Plant species richness	0.14	0.07	2.1	0.039	0.23
**Vertical structural stratification ~**					
Plant species richness	0.03	0.01	2.2	0.028	0.18
Plant biomass	0.11	0.02	4.9	< 0.001	0.43
**Horizontal structural variation ~**					
Plant biomass	-0.05	0.01	-4.9	< 0.001	-0.44
**Arthropod abundance ~**					
Functional diversity	*0.08*	*0.04*	*2.0*	*0.051*	*0.20*
Trait composition 2	0.05	0.02	2.9	0.004	0.25
**Arthropod species richness ~**					
Functional diversity	0.04	0.02	2.1	0.034	0.15
Plant biomass	0.11	0.04	3.0	0.003	0.20
Arthropod abundance	0.43	0.06	7.5	< 0.001	0.67

Variances

Variable	Estimate	SE	*z*	*P* (bootstr.)	Stand. Est.
Plant species richness	0.24	0.03	7.3	< 0.001	1.00
Functional diversity	0.20	0.03	7.1	< 0.001	0.53
Trait composition 2	1.34	0.25	5.3	< 0.001	0.96
Plant biomass	0.08	0.01	7.1	< 0.001	0.95
Vertical structural stratification	0.00	0.00	8.3	< 0.001	0.75
Horizontal structural variation	0.00	0.00	6.9	< 0.001	0.80
Arthropod abundance	0.06	0.01	5.3	< 0.001	0.91
Arthropod species richness	0.01	0.00	8.3	< 0.001	0.46

Covariances

Variables	Estimate	SE	*z*	*P* (bootstr.)	Stand. Est.
**Vertical structural stratification ~~**					
Horizontal structural variation	0.00	0.00	-5.0	< 0.001	-0.50
**Trait composition 2 ~~**					
Plant biomass	-0.11	0.04	-2.6	0.010	-0.35

R²

Variable	Estimate
Functional diversity	0.47
Trait composition 2	0.04
Plant biomass	0.05
Vertical structural stratification	0.25

Horizontal structural variation	0.20
Arthropod abundance	0.09
Arthropod species richness	0.54

Non-significant ($P > 0.05$ based on 1,000 bootstrap draws) predictors are printed in italics. RMSEA = Root mean square error of approximation.

Supplementary Table 9. Path model output for biodiversity effects on *parasitoid* species richness in the Jena *grassland experiment*.

**Model**					
AICc initial model	437.2				
AICc minimal model	392.2				
Number of observations	92				
Chi-square	27.2				
P (Chi-square)	0.166				
P (Bollen-Stine Bootstrap)	0.302				
Degrees of freedom	21				
RMSEA	0.056				
95% CI (RMSEA)	0 - 0.111				
P (RMSEA)	0.396				
**Regressions**					
Respone ~ Predictor	Estimate	SE	*z*	*P* (bootstr.)	Stand. Est.
**Functional diversity ~**					
Plant species richness	0.86	0.08	11.1	< 0.001	0.69
**Trait composition 2 ~**					
Plant species richness	*-0.46*	*0.26*	*-1.8*	*0.080*	*-0.19*
**Plant biomass ~**					
Plant species richness	0.14	0.07	2.0	0.041	0.23
**Vertical structural stratification ~**					
Plant species richness	0.03	0.01	2.7	0.007	0.21
Plant biomass	0.09	0.02	4.4	< 0.001	0.38
**Horizontal structural variation ~**					
Plant biomass	-0.04	0.01	-4.2	< 0.001	-0.40
**Arthropod abundance ~**					
Functional diversity	0.34	0.09	3.7	< 0.001	0.34
Trait composition 2	0.20	0.05	3.9	< 0.001	0.40
Plant biomass	0.69	0.23	3.0	0.003	0.33
Horizontal structural variation	5.32	2.17	2.5	0.014	0.27
**Arthropod species richness ~**					
Arthropod abundance	0.68	0.05	15.0	< 0.001	0.88
**Variances**					
Variable	Estimate	SE	*z*	*P* (bootstr.)	Stand. Est.
Plant species richness	0.24	0.03	7.2	< 0.001	1.00
Functional diversity	0.20	0.03	7.4	< 0.001	0.53
Trait composition 1	2.76	0.39	7.0	< 0.001	1.00
Trait composition 2	1.34	0.26	5.2	< 0.001	0.96
Plant biomass	0.08	0.01	7.1	< 0.001	0.95
Vertical structural stratification	0.00	0.00	8.4	< 0.001	0.78
Horizontal structural variation	0.00	0.00	7.2	< 0.001	0.84
Arthropod abundance	0.25	0.03	7.8	< 0.001	0.69
Arthropod species richness	0.05	0.01	5.0	< 0.001	0.22
**Covariances**					
Variables	Estimate	SE	*z*	*P* (bootstr.)	Stand. Est.
**Vertical structural stratification ~~**					
Horizontal structural variation	0.00	0.00	-5.2	< 0.001	-0.51
Trait composition 1	0.05	0.01	4.7	< 0.001	0.43
**Horizontal structural variation ~~**					
Trait composition 1	-0.02	0.01	-4.0	< 0.001	-0.44
**Trait composition 2 ~~**					
Plant biomass	-0.11	0.05	-2.5	0.012	-0.35
R²					
Variable	Estimate				

Functional diversity	0.47
Trait composition 2	0.04
Plant biomass	0.05
Vertical structural stratification	0.22
Horizontal structural variation	0.16
Arthropod abundance	0.31
Arthropod species richness	0.78

Non-significant ($P > 0.05$ based on 1,000 bootstrap draws) predictors are printed in italics. RMSEA = Root mean square error of approximation.

Supplementary Table 10. Alternative path model for biodiversity effects on *overall arthropod* species richness in the BEF-China *forest experiment, with the relationship between arthropod abundance and species richness fitted as covariance*.

**Model**					
AICc initial model	2789.5				
AICc minimal model	591.7				
Number of observations	46				
Chi-square	4.96				
P (Chi-square)	0.838				
P (Bollen-Stine Bootstrap)	0.849				
Degrees of freedom	9				
RMSEA	0				
95% CI (RMSEA)	0 - 0.097				
P (RMSEA)	0.881				

**Regressions**					
Respone ~ Predictor	Estimate	SE	*z*	*P* (bootstr.)	Stand. Est.
**Functional diversity ~**					
Tree species richness	0.353	0.049	7.3	< 0.001	0.69
**Tree biomass ~**					
Tree species richness	0.737	0.209	3.5	< 0.001	0.47
**Vertical structural stratification ~**					
Tree biomass	0.647	0.119	5.4	< 0.001	0.71
**Horizontal structural variation ~**					
Tree species richness	-0.440	0.167	-2.6	0.009	-0.34
Tree biomass	0.416	0.120	3.5	0.001	0.50
**Arthropod abundance ~**					
Functional diversity	0.264	0.075	3.5	< 0.001	0.38
Vertical structural stratification	0.074	0.030	2.4	0.015	0.30
**Arthropod species richness ~**					
Functional diversity	0.234	0.048	4.9	< 0.001	0.51
Tree biomass	0.051	0.017	2.9	0.003	0.34
Horizontal structural variation	-0.060	0.021	-2.9	0.004	-0.33

**Variances**					
Variable	Estimate	SE	*z*	*P* (bootstr.)	Stand. Est.
Tree species richness	0.835	0.174	4.8	< 0.001	1.00
Functional diversity	0.117	0.030	3.8	< 0.001	0.53
Tree biomass	1.609	0.287	5.6	< 0.001	0.78
Vertical structural stratification	0.834	0.226	3.7	< 0.001	0.49
Horizontal structural variation	1.108	0.181	6.1	< 0.001	0.79
Arthropod abundance	0.080	0.020	4.0	< 0.001	0.75
Arthropod species richness	0.024	0.005	5.2	< 0.001	0.50

**Covariances**					
Variables	Estimate	SE	*z*	*P* (bootstr.)	Stand. Est.
**Functional diversity ~~**					
Tree biomass	-0.14	0.06	-2.5	0.013	-0.32
**Arthropod species richness ~~**					
Arthropod abundance	*0.02*	*0.01*	*1.7*	*0.088*	*0.37*

R^2	
Variable	Estimate
Functional diversity	0.47
Tree biomass	0.22
Vertical structural stratification	0.51
Horizontal structural variation	0.21
Arthropod abundance	0.25
Arthropod species richness	0.50

Non-significant (*P* > 0.05 based on 1,000 bootstrap draws) predictors are printed in italics. RMSEA = Root mean square error of approximation.

Supplementary Table 11. Alternative path model for biodiversity effects on *herbivore* species richness in the BEF-China *forest experiment, with the relationship between arthropod abundance and species richness fitted as covariance*.

**Model**					
AICc initial model	2911.3				
AICc minimal model	706.0				
Number of observations	46				
Chi-square	6.6				
P (Chi-square)	0.828				
P (Bollen-Stine Bootstrap)	0.792				
Degrees of freedom	11				
RMSEA	0				
95% CI (RMSEA)	0 - 0.094				
P (RMSEA)	0.878				
**Regressions**					
Respone ~ Predictor	Estimate	SE	*z*	*P* (bootstr.)	Stand. Est.
**Functional diversity ~**					
Tree species richness	0.35	0.05	7.4	< 0.001	0.69
**Tree biomass ~**					
Tree species richness	0.74	0.21	3.5	< 0.001	0.47
**Vertical structural stratification ~**					
Tree biomass	0.65	0.12	5.4	< 0.001	0.71
**Horizontal structural variation ~**					
Tree species richness	-0.44	0.17	-2.5	0.011	-0.34
Tree biomass	0.42	0.13	3.3	0.001	0.50
**Arthropod abundance ~**					
Tree species richness	0.22	0.08	3.0	0.003	0.35
**Arthropod species richness ~**					
Tree species richness	0.25	0.05	4.7	< 0.001	0.54
Horizontal structural variation	-0.09	0.04	-2.3	0.021	-0.26
**Variances**					
Variable	Estimate	SE	*z*	*P* (bootstr.)	Stand. Est.
Tree species richness	0.84	0.17	4.9	< 0.001	1.00
Functional diversity	0.12	0.03	4.1	< 0.001	0.53
Tree biomass	1.61	0.28	5.7	< 0.001	0.78
Vertical structural stratification	0.83	0.22	3.7	< 0.001	0.49
Horizontal structural variation	1.11	0.18	6.3	< 0.001	0.79
Arthropod abundance	0.30	0.09	3.5	< 0.001	0.88
Arthropod species richness	0.11	0.03	4.3	< 0.001	0.61
**Covariances**					
Variables	Estimate	SE	*z*	*P* (bootstr.)	Stand. Est.
**Functional diversity ~~**					
Tree biomass	-0.14	0.06	-2.4	0.017	-0.32
**Arthropod species richness ~~**					
Arthropod abundance	0.09	0.04	2.5	0.012	0.50
R²					
Variable	Estimate				
Functional diversity	0.47				
Tree biomass	0.22				
Vertical structural stratification	0.51				
Horizontal structural variation	0.21				
Arthropod abundance	0.12				
Arthropod species richness	0.39				

Non-significant (P > 0.05 based on 1,000 bootstrap draws) predictors are printed in italics. RMSEA = Root mean square error of approximation.

Supplementary Table 12. Alternative path model for biodiversity effects on *predator* species richness in the BEF-China *forest experiment, with the relationship between arthropod abundance and species richness fitted as covariance*.

**Model**	
AICc initial model	2799.5
AICc minimal model	880.8
Number of observations	46
Chi-square	14.3
P (Chi-square)	0.889
P (Bollen-Stine Bootstrap)	0.843
Degrees of freedom	22
RMSEA	0
95% CI (RMSEA)	0 - 0.058
P (RMSEA)	0.938

Regressions

Respone ~ Predictor	Estimate	SE	*z*	*P* (bootstr.)	Stand. Est.
**Functional diversity ~**					
Tree species richness	0.35	0.05	7.7	< 0.001	0.69
**Tree biomass ~**					
Tree species richness	0.74	0.22	3.4	0.001	0.47
**Vertical structural stratification ~**					
Tree biomass	0.65	0.12	5.3	< 0.001	0.71
**Horizontal structural variation ~**					
Tree species richness	-0.44	0.18	-2.5	0.013	-0.34
Tree biomass	0.42	0.12	3.5	< 0.001	0.50
**Arthropod abundance ~**					
Tree biomass	0.16	0.05	2.9	0.003	0.53
Trait composition 1	-0.07	0.02	-2.9	0.004	-0.24
Trait composition 2	0.09	0.04	2.0	0.047	0.22
Vertical structural stratification	-0.25	0.06	-4.3	< 0.001	-0.78
**Arthropod species richness ~**					
Functional diversity	0.14	0.05	2.8	0.004	0.24
Tree biomass	0.11	0.03	3.4	0.001	0.59
Vertical structural stratification	-0.16	0.04	-4.1	< 0.001	-0.76

Variances

Variable	Estimate	SE	*z*	*P* (bootstr.)	Stand. Est.
Tree species richness	0.84	0.18	4.6	< 0.001	1.00
Functional diversity	0.12	0.03	4.0	< 0.001	0.53
Trait composition 1	2.03	0.67	3.0	0.003	1.00
Trait composition 2	1.16	0.50	2.3	0.021	1.00
Tree biomass	1.61	0.29	5.6	< 0.001	0.78
Vertical structural stratification	0.83	0.24	3.6	< 0.001	0.49
Horizontal structural variation	1.11	0.17	6.4	< 0.001	0.79
Arthropod abundance	0.10	0.02	5.8	< 0.001	0.59
Arthropod species richness	0.05	0.01	3.9	< 0.001	0.66

Covariances

Variables	Estimate	SE	*z*	*P* (bootstr.)	Stand. Est.
**Functional diversity ~~**					
Tree biomass	-0.14	0.06	-2.5	0.012	-0.32
**Arthropod species richness ~~**					
Arthropod abundance	0.05	0.01	3.9	< 0.001	0.77

R^2

Variable	Estimate
Functional diversity	0.47
Tree biomass	0.22

Vertical structural stratification	0.51
Horizontal structural variation	0.21
Arthropod abundance	0.41
Arthropod species richness	0.34

Non-significant ($P > 0.05$ based on 1,000 bootstrap draws) predictors are printed in italics. RMSEA = Root mean square error of approximation.

Supplementary Table 13. Alternative path model for biodiversity effects on *parasitoid* species richness in the BEF-China *forest experiment, with the relationship between arthropod abundance and species richness fitted as covariance*.

**Model**					
AICc initial model	2917.7				
AICc minimal model	882.9				
Number of observations	46				
Chi-square	10.1				
P (Chi-square)	0.927				
P (Bollen-Stine Bootstrap)	0.892				
Degrees of freedom	18				
RMSEA	0				
95% CI (RMSEA)	0 - 0.041				
P (RMSEA)	0.958				
**Regressions**					
Respone ~ Predictor	Estimate	SE	*z*	*P* (bootstr.)	Stand. Est.
**Functional diversity ~**					
Tree species richness	0.35	0.05	7.9	< 0.001	0.69
**Tree biomass ~**					
Tree species richness	0.74	0.22	3.4	0.001	0.47
**Vertical structural stratification ~**					
Tree biomass	0.65	0.12	5.3	< 0.001	0.71
**Horizontal structural variation ~**					
Tree species richness	-0.44	0.17	-2.6	0.009	-0.34
Tree biomass	0.42	0.12	3.6	< 0.001	0.50
**Arthropod abundance ~**					
Trait composition 1	-0.10	0.05	-2.1	0.035	-0.19
Vertical structural stratification	0.21	0.06	3.5	< 0.001	0.38
**Arthropod species richness ~**					
Vertical structural stratification	0.19	0.05	3.7	< 0.001	0.49
**Variances**					
Variable	Estimate	SE	*z*	*P* (bootstr.)	Stand. Est.
Tree species richness	0.84	0.18	4.7	< 0.001	1.00
Functional diversity	0.12	0.03	4.2	< 0.001	0.53
Trait composition 1	2.03	0.67	3.0	0.002	1.00
Tree biomass	1.61	0.28	5.7	< 0.001	0.78
Vertical structural stratification	0.83	0.23	3.6	< 0.001	0.49
Horizontal structural variation	1.11	0.19	5.8	< 0.001	0.79
Arthropod abundance	0.44	0.10	4.3	< 0.001	0.82
Arthropod species richness	0.19	0.05	4.2	< 0.001	0.76
**Covariances**					
Variables	Estimate	SE	*z*	*P* (bootstr.)	Stand. Est.
**Functional diversity ~~**					
Tree biomass	-0.14	0.06	-2.5	0.012	-0.32
**Arthropod species richness ~~**					
Arthropod abundance	0.24	0.07	3.6	< 0.001	0.81
R²					
Variable	Estimate				
Functional diversity	0.47				
Tree biomass	0.22				
Vertical structural stratification	0.51				
Horizontal structural variation	0.21				
Arthropod abundance	0.18				
Arthropod species richness	0.24				

Non-significant ($P > 0.05$ based on 1,000 bootstrap draws) predictors are printed in italics.
RMSEA = Root mean square error of approximation.

Supplementary Table 14. Alternative path model for biodiversity effects on *overall arthropod* species richness in the Jena *grassland experiment, with the relationship between arthropod abundance and species richness fitted as covariance*.

**Model**	
AICc initial model	168.9
AICc minimal model	99.6
Number of observations	92
Chi-square	23.5
P (Chi-square)	0.215
P (Bollen-Stine Bootstrap)	0.284
Degrees of freedom	19
RMSEA	0.051
95% CI (RMSEA)	0 - 0.110
P (RMSEA)	0.45

Regressions

Respone ~ Predictor	Estimate	SE	*z*	*P* (bootstr.)	Stand. Est.
**Functional diversity ~**					
Plant species richness	0.86	0.07	12.1	< 0.001	0.69
**Trait composition 2 ~**					
Tree species richness	*-0.46*	*0.27*	*-1.7*	*0.092*	*-0.19*
**Plant biomass ~**					
Tree species richness	0.14	0.07	2.1	0.037	0.23
**Vertical structural stratification ~**					
Plant species richness	0.03	0.01	2.7	0.007	0.21
Plant biomass	0.09	0.02	4.8	< 0.001	0.38
**Horizontal structural variation ~**					
Plant biomass	-0.04	0.01	-4.4	< 0.001	-0.40
**Arthropod abundance ~**					
Functional diversity	0.08	0.04	2.1	0.033	0.20
Vertical structural stratification	-0.76	0.31	-2.5	0.014	-0.23
**Arthropod species richness ~**					
Functional diversity	0.09	0.02	4.2	< 0.001	0.37
Trait composition 1	-0.02	0.01	-2.7	0.007	-0.21
Trait composition 2	0.02	0.01	2.3	0.022	0.16
Plant biomass	0.08	0.04	2.1	0.037	0.15

Variances

Variable	Estimate	SE	*z*	*P* (bootstr.)	Stand. Est.
Plant species richness	0.24	0.03	7.3	< 0.001	1.00
Functional diversity	0.20	0.03	7.5	< 0.001	0.53
Trait composition 1	2.76	0.40	6.8	< 0.001	1.00
Trait composition 2	1.34	0.25	5.3	< 0.001	0.96
Plant biomass	0.08	0.01	7.0	< 0.001	0.95
Vertical structural stratification	0.00	0.00	8.0	< 0.001	0.78
Horizontal structural variation	0.00	0.00	6.8	< 0.001	0.84
Arthropod abundance	0.05	0.01	5.3	< 0.001	0.93
Arthropod species richness	0.02	0.00	6.1	< 0.001	0.79

Covariances

Variables	Estimate	SE	*z*	*P* (bootstr.)	Stand. Est.
**Vertical structural stratification ~~**					
Horizontal structural variation	0.00	0.00	-5.1	< 0.001	-0.51
Trait composition 1	0.05	0.01	4.7	< 0.001	0.43
**Horizontal structural variation ~~**					
Trait composition 1	-0.02	0.01	-3.9	< 0.001	-0.44
**Plant biomass ~~**					
Trait composition 2	-0.11	0.04	-2.8	0.006	-0.35
**Arthropod species richness ~~**					

Arthropod abundance	0.02	0.01	4.2	< 0.001	0.69

R^2

Variable	Estimate
Functional diversity	0.47
Trait composition 2	0.04
Plant biomass	0.05
Vertical structural stratification	0.19
Horizontal structural variation	0.16
Arthropod abundance	0.08
Arthropod species richness	0.21

Non-significant (*P* > 0.05 based on 1,000 bootstrap draws) predictors are printed in italics. RMSEA = Root mean square error of approximation.

Supplementary Table 15. Alternative path model for biodiversity effects on *herbivore* species richness in the Jena *grassland experiment, with the relationship between arthropod abundance and species richness fitted as covariance*.

**Model**					
AICc initial model	410.1				
AICc minimal model	338.8				
Number of observations	92				
Chi-square	25.3				
P (Chi-square)	0.188				
P (Bollen-Stine Bootstrap)	0.238				
Degrees of freedom	20				
RMSEA	0.054				
95% CI (RMSEA)	0 - 0.110				
P (RMSEA)	0.421				
**Regressions**					
Respone ~ Predictor	Estimate	SE	*z*	*P* (bootstr.)	Stand. Est.
**Functional diversity ~**					
Plant species richness	0.86	0.08	11.4	< 0.001	0.69
**Trait composition 2 ~**					
Tree species richness	*-0.46*	*0.27*	*-1.7*	*0.085*	*-0.19*
**Plant biomass ~**					
Tree species richness	0.14	0.07	2.0	0.041	0.23
**Vertical structural stratification ~**					
Plant species richness	0.03	0.01	2.9	0.004	0.21
Plant biomass	0.09	0.02	4.6	< 0.001	0.38
**Horizontal structural variation ~**					
Plant biomass	-0.04	0.01	-4.3	< 0.001	-0.40
**Arthropod abundance ~**					
Functional diversity	0.22	0.07	3.0	0.002	0.27
Vertical structural stratification	-2.74	0.63	-4.4	< 0.001	-0.41
**Arthropod species richness ~**					
Plant species richness	0.13	0.04	3.0	0.003	0.28
Trait composition 1	-0.04	0.01	-3.3	0.001	-0.26
Trait composition 2	0.05	0.02	3.2	0.001	0.22
**Variances**					
Variable	Estimate	SE	*z*	*P* (bootstr.)	Stand. Est.
Plant species richness	0.24	0.03	7.2	< 0.001	1.00
Functional diversity	0.20	0.03	6.8	< 0.001	0.53
Trait composition 1	2.76	0.40	7.0	< 0.001	1.00
Trait composition 2	1.34	0.25	5.3	< 0.001	0.96
Plant biomass	0.08	0.01	6.7	< 0.001	0.95
Vertical structural stratification	0.00	0.00	8.4	< 0.001	0.78
Horizontal structural variation	0.00	0.00	6.9	< 0.001	0.84
Arthropod abundance	0.19	0.04	5.2	< 0.001	0.80
Arthropod species richness	0.06	0.01	4.6	< 0.001	0.82
**Covariances**					
Variables	Estimate	SE	*z*	*P* (bootstr.)	Stand. Est.
**Vertical structural stratification ~~**					
Horizontal structural variation	0.00	0.00	-4.9	< 0.001	-0.51
Trait composition 1	0.05	0.01	4.6	< 0.001	0.43
**Horizontal structural variation ~~**					
Trait composition 1	-0.02	0.01	-3.9	< 0.001	-0.44
**Trait composition 2 ~~**					
Plant biomass	-0.11	0.04	-2.6	0.010	-0.35
**Arthropod species richness ~~**					
Arthropod abundance	0.07	0.02	3.3	0.001	0.65

R^2

Variable	Estimate
Functional diversity	0.47
Trait composition 2	0.04
Plant biomass	0.05
Vertical structural stratification	0.22
Horizontal structural variation	0.16
Arthropod abundance	0.20
Arthropod species richness	0.18

Non-significant ($P > 0.05$ based on 1,000 bootstrap draws) predictors are printed in italics. RMSEA = Root mean square error of approximation.

Supplementary Table 16. Alternative path model for biodiversity effects on *predator* species richness in the Jena *grassland experiment, with the relationship between arthropod abundance and species richness fitted as covariance*.

**Model**					
AICc initial model	199.4				
AICc minimal model	-171.0				
Number of observations	92				
Chi-square	21.3				
P (Chi-square)	0.095				
P (Bollen-Stine Bootstrap)	0.170				
Degrees of freedom	14				
RMSEA	0.075				
95% CI (RMSEA)	0 - 0.136				
P (RMSEA)	0.239				

**Regressions**					
Respone ~ Predictor	Estimate	SE	*z*	*P* (bootstr.)	Stand. Est.
**Functional diversity ~**					
Plant species richness	0.86	0.08	11.0	< 0.001	0.69
**Plant biomass ~**					
Plant species richness	0.14	0.07	2.0	0.043	0.23
**Vertical structural stratification ~**					
Plant species richness	0.03	0.01	2.7	0.006	0.21
Plant biomass	0.09	0.02	4.6	< 0.001	0.38
**Horizontal structural variation ~**					
Plant biomass	-0.04	0.01	-4.2	< 0.001	-0.40
**Arthropod abundance ~**					
Plant species richness	0.09	0.05	2.0	0.049	0.17
Vertical structural stratification	-0.78	0.28	-2.8	0.006	-0.23
Trait composition 1	0.03	0.01	2.4	0.016	0.21
**Arthropod species richness ~**					
Functional diversity	0.06	0.02	2.9	0.003	0.23
Plant biomass	0.09	0.04	2.2	0.028	0.17

**Variances**					
Variable	Estimate	SE	*z*	*P* (bootstr.)	Stand. Est.
Plant species richness	0.24	0.03	7.5	< 0.001	1.00
Functional diversity	0.20	0.03	7.2	< 0.001	0.53
Trait composition 1	2.76	0.40	6.9	< 0.001	1.00
Plant biomass	0.08	0.01	6.9	< 0.001	0.95
Vertical structural stratification	0.00	0.00	8.1	< 0.001	0.78
Horizontal structural variation	0.00	0.00	7.1	< 0.001	0.84
Arthropod abundance	0.06	0.01	5.1	< 0.001	0.93
Arthropod species richness	0.02	0.01	4.5	< 0.001	0.91

**Covariances**					
Variables	Estimate	SE	*z*	*P* (bootstr.)	Stand. Est.
**Vertical structural stratification ~~**					
Horizontal structural variation	0.00	0.00	-4.9	< 0.001	-0.51
Trait composition 1	0.05	0.01	4.8	< 0.001	0.43
**Horizontal structural variation ~~**					
Trait composition 1	-0.02	0.01	-3.9	< 0.001	-0.44
**Arthropod species richness ~~**					
Arthropod abundance	0.03	0.01	3.5	< 0.001	0.70

R^2	
Variable	Estimate
Functional diversity	0.47
Plant biomass	0.05

Vertical structural stratification	0.22
Horizontal structural variation	0.16
Arthropod abundance	0.07
Arthropod species richness	0.09

Non-significant ($P > 0.05$ based on 1,000 bootstrap draws) predictors are printed in italics. RMSEA = Root mean square error of approximation.

Supplementary Table 17. Alternative path model for biodiversity effects on *parasitoid* species richness in the Jena *grassland experiment, with the relationship between arthropod abundance and species richness fitted as covariance*.

**Model**					
AICc initial model	437.2				
AICc minimal model	403.6				
Number of observations	92				
Chi-square	22.7				
P (Chi-square)	0.161				
P (Bollen-Stine Bootstrap)	0.271				
Degrees of freedom	17				
RMSEA	0.060				
95% CI (RMSEA)	0 - 0.119				
P (RMSEA)	0.363				
**Regressions**					
Respone ~ Predictor	Estimate	SE	*z*	*P* (bootstr.)	Stand. Est.
**Functional diversity ~**					
Plant species richness	0.86	0.08	11.3	< 0.001	0.69
**Trait composition 2 ~**					
Plant species richness	*-0.46*	*0.27*	*-1.7*	*0.091*	*-0.19*
**Plant biomass ~**					
Plant species richness	0.14	0.07	2.0	0.048	0.23
**Vertical structural stratification ~**					
Plant species richness	0.03	0.01	2.6	0.009	0.21
Plant biomass	0.09	0.02	4.5	< 0.001	0.38
**Horizontal structural variation ~**					
Plant biomass	-0.04	0.01	-4.3	< 0.001	-0.40
**Arthropod abundance ~**					
Functional diversity	0.34	0.09	3.9	< 0.001	0.34
Trait composition 2	0.20	0.05	4.3	< 0.001	0.40
Plant biomass	0.69	0.23	3.0	0.002	0.33
Horizontal structural variation	5.32	2.10	2.5	0.011	0.27
**Arthropod species richness ~**					
Functional diversity	0.25	0.07	3.3	0.001	0.32
Trait composition 2	0.10	0.04	2.4	0.016	0.26
Plant biomass	0.38	0.18	2.1	0.038	0.23
Horizontal structural variation	4.17	1.56	2.7	0.007	0.28
**Variances**					
Variable	Estimate	SE	*z*	*P* (bootstr.)	Stand. Est.
Plant species richness	0.24	0.03	7.1	< 0.001	1.00
Functional diversity	0.20	0.03	7.0	< 0.001	0.53
Trait composition 1	2.76	0.39	7.1	< 0.001	1.00
Trait composition 2	1.34	0.25	5.4	< 0.001	0.96
Plant biomass	0.08	0.01	7.0	< 0.001	0.95
Vertical structural stratification	0.00	0.00	8.3	< 0.001	0.78
Horizontal structural variation	0.00	0.00	7.2	< 0.001	0.84
Arthropod abundance	0.25	0.03	7.7	< 0.001	0.69
Arthropod species richness	0.17	0.03	5.6	< 0.001	0.78
**Covariances**					
Variables	Estimate	SE	*z*	*P* (bootstr.)	Stand. Est.
**Vertical structural stratification ~~**					
Horizontal structural variation	0.00	0.00	-5.0	< 0.001	-0.51
Trait composition 1	0.05	0.01	4.7	< 0.001	0.43
**Horizontal structural variation ~~**					
Trait composition 1	-0.02	0.01	-3.9	< 0.001	-0.44
**Trait composition 2 ~~**					

Plant biomass	-0.11	0.04	-2.7	0.007	-0.35
**Arthropod species richness ~~**					
Arthropod abundance	0.18	0.03	6.3	< 0.001	0.85

R²

Variable	Estimate
Functional diversity	0.47
Trait composition 2	0.04
Plant biomass	0.05
Vertical structural stratification	0.22
Horizontal structural variation	0.16
Arthropod abundance	0.31
Arthropod species richness	0.22

Non-significant ($P > 0.05$ based on 1,000 bootstrap draws) predictors are printed in italics. RMSEA = Root mean square error of approximation.

Supplementary Table 18. Alternative path model for biodiversity effects on *overall arthropod* species richness in the BEF-China *forest experiment, with effects of arthropod abundance factored out by rarefaction*.

**Model**					
AICc initial model	1175.2				
AICc minimal model	808.9				
Number of observations	46				
Chi-square	7.9				
P (Chi-square)	0.548				
P (Bollen-Stine Bootstrap)	0.555				
Degrees of freedom	9				
RMSEA	0				
95% CI (RMSEA)	0 - 0.151				
P (RMSEA)	0.633				
**Regressions**					
Respone ~ Predictor	Estimate	SE	*z*	*P* (bootstr.)	Stand. Est.
**Functional diversity ~**					
Plant species richness	0.35	0.04	8.1	< 0.001	0.69
**Plant biomass ~**					
Plant species richness	0.74	0.22	3.4	0.001	0.47
**Vertical structural stratification ~**					
Plant biomass	0.65	0.12	5.6	< 0.001	0.71
**Horizontal structural variation ~**					
Plant species richness	-0.44	0.16	-2.7	0.008	-0.34
Plant biomass	0.42	0.12	3.4	0.001	0.50
**Variances**					
Variable	Estimate	SE	*z*	*P* (bootstr.)	Stand. Est.
Plant species richness	0.84	0.18	4.8	< 0.001	1.00
Functional diversity	0.12	0.03	3.9	< 0.001	0.53
Plant biomass	1.61	0.29	5.5	< 0.001	0.78
Vertical structural stratification	0.83	0.22	3.8	< 0.001	0.49
Horizontal structural variation	1.11	0.18	6.2	< 0.001	0.79
Arthropod species richness	6.49	1.72	3.8	< 0.001	1.00
**Covariances**					
Variables	Estimate	SE	*z*	*P* (bootstr.)	Stand. Est.
**Functional diversity ~~**					
Plant biomass	-0.14	0.06	-2.5	0.014	-0.32
R^2					
Variable	Estimate				
Functional diversity	0.47				
Plant biomass	0.22				
Vertical structural stratification	0.51				
Horizontal structural variation	0.21				

Non-significant ($P > 0.05$ based on 1,000 bootstrap draws) predictors are printed in italics.
RMSEA = Root mean square error of approximation.

Supplementary Table 19. Alternative path model for biodiversity effects on *herbivore* species richness in the BEF-China *forest experiment, with effects of arthropod abundance factored out by rarefaction*.

**Model**					
AICc initial model	944.9				
AICc minimal model	602.6				
Number of observations	46				
Chi-square	7.4				
P (Chi-square)	0.600				
P (Bollen-Stine Bootstrap)	0.599				
Degrees of freedom	9				
RMSEA	0				
95% CI (RMSEA)	0 - 0.143				
P (RMSEA)	0.681				
**Regressions**					
Respone ~ Predictor	Estimate	SE	*z*	*P* (bootstr.)	Stand. Est.
**Functional diversity ~**					
Plant species richness	0.35	0.04	8.0	< 0.001	0.69
**Plant biomass ~**					
Plant species richness	0.74	0.23	3.2	0.001	0.47
**Vertical structural stratification ~**					
Plant biomass	0.65	0.12	5.4	< 0.001	0.71
**Horizontal structural variation ~**					
Plant species richness	-0.44	0.18	-2.5	0.013	-0.34
Plant biomass	0.42	0.12	3.4	0.001	0.50
**Variances**					
Variable	Estimate	SE	*z*	*P* (bootstr.)	Stand. Est.
Plant species richness	0.84	0.18	4.8	< 0.001	1.00
Functional diversity	0.12	0.03	4.1	< 0.001	0.53
Plant biomass	1.61	0.29	5.5	< 0.001	0.78
Vertical structural stratification	0.83	0.23	3.7	< 0.001	0.49
Horizontal structural variation	1.11	0.18	6.1	< 0.001	0.79
Arthropod species richness	0.07	0.03	2.2	0.030	1.00
**Covariances**					
Variables	Estimate	SE	*z*	*P* (bootstr.)	Stand. Est.
**Functional diversity ~~**					
Plant biomass	-0.14	0.06	-2.5	0.011	-0.32
**R^2**					
Variable	Estimate				
Functional diversity	0.47				
Plant biomass	0.22				
Vertical structural stratification	0.51				
Horizontal structural variation	0.21				

Non-significant ($P > 0.05$ based on 1,000 bootstrap draws) predictors are printed in italics. RMSEA = Root mean square error of approximation.

Supplementary Table 20. Alternative path model for biodiversity effects on *predator* species richness in the BEF-China *forest experiment, with effects of arthropod abundance factored out by rarefaction*.

Model					
AICc initial model	1029.6				
AICc minimal model	857.9				
Number of observations	46				
Chi-square	7.2				
P (Chi-square)	0.928				
P (Bollen-Stine Bootstrap)	0.880				
Degrees of freedom	14				
RMSEA	0				
95% CI (RMSEA)	0 - 0.044				
P (RMSEA)	0.955				
**Regressions**					
Respone ~ Predictor	Estimate	SE	*z*	*P* (bootstr.)	Stand. Est.
**Functional diversity ~**					
Plant species richness	0.35	0.05	7.8	< 0.001	0.69
**Plant biomass ~**					
Plant species richness	0.74	0.22	3.4	0.001	0.47
**Vertical structural stratification ~**					
Plant biomass	0.65	0.13	5.2	< 0.001	0.71
**Horizontal structural variation ~**					
Plant species richness	-0.44	0.18	-2.5	0.012	-0.34
Plant biomass	0.42	0.12	3.3	0.001	0.50
**Arthropod species richness ~**					
Trait composition 1	0.16	0.09	1.7	0.085	0.32
**Variances**					
Variable	Estimate	SE	*z*	*P* (bootstr.)	Stand. Est.
Plant species richness	0.84	0.17	4.8	< 0.001	1.00
Functional diversity	0.12	0.03	3.9	< 0.001	0.53
Trait composition 1	2.03	0.66	3.1	0.002	1.00
Plant biomass	1.61	0.27	5.9	< 0.001	0.78
Vertical structural stratification	0.83	0.23	3.6	< 0.001	0.49
Horizontal structural variation	1.11	0.18	6.0	< 0.001	0.79
Arthropod species richness	0.45	0.09	4.9	< 0.001	0.90
**Covariances**					
Variables	Estimate	SE	*z*	*P* (bootstr.)	Stand. Est.
**Functional diversity ~~**					
Plant biomass	-0.14	0.06	-2.4	0.018	-0.32
R²					
Variable	Estimate				
Functional diversity	0.47				
Plant biomass	0.22				
Vertical structural stratification	0.51				
Horizontal structural variation	0.21				
Arthropod species richness	0.10				

Non-significant (*P* > 0.05 based on 1,000 bootstrap draws) predictors are printed in italics. RMSEA = Root mean square error of approximation.

Supplementary Table 21. Alternative path model for biodiversity effects on *overall arthropod* species richness in the Jena *grassland experiment, with effects of arthropod abundance factored out by rarefaction*.

**Model**	
AICc initial model	787.6
AICc minimal model	780.2
Number of observations	92
Chi-square	22.4
P (Chi-square)	0.072
P (Bollen-Stine Bootstrap)	0.122
Degrees of freedom	14
RMSEA	0.081
95% CI (RMSEA)	0 - 0.140
P (RMSEA)	0.122

Regressions

Respone ~ Predictor	Estimate	SE	*z*	*P* (bootstr.)	Stand. Est.
**Functional diversity ~**					
Plant species richness	0.86	0.08	11.4	< 0.001	0.69
**Trait composition 2 ~**					
Plant species richness	*-0.46*	*0.27*	*-1.7*	*0.093*	*-0.19*
**Plant biomass ~**					
Plant species richness	0.14	0.07	2.1	0.037	0.23
**Vertical structural stratification ~**					
Plant species richness	0.03	0.01	2.7	0.006	0.21
Plant biomass	0.09	0.02	4.5	< 0.001	0.38
**Horizontal structural variation ~**					
Plant biomass	-0.04	0.01	-4.1	< 0.001	-0.40
**Arthropod species richness ~**					
Functional diversity	1.61	0.76	2.1	0.035	0.22
Trait composition 1	*-0.49*	*0.26*	*-1.9*	*0.064*	*-0.18*
Plant biomass	3.42	1.45	2.4	0.018	0.22
Vertical structural stratification	*13.13*	*7.27*	*1.8*	*0.071*	*0.21*

Variances

Variable	Estimate	SE	*z*	*P* (bootstr.)	Stand. Est.
Plant species richness	0.24	0.03	7.1	< 0.001	1.00
Functional diversity	0.20	0.03	7.1	< 0.001	0.53
Trait composition 1	2.76	0.39	7.0	< 0.001	1.00
Trait composition 2	1.34	0.27	5.0	< 0.001	0.96
Plant biomass	0.08	0.01	7.2	< 0.001	0.95
Vertical structural stratification	0.00	0.00	8.8	< 0.001	0.78
Horizontal structural variation	0.00	0.00	7.2	< 0.001	0.84
Arthropod species richness	15.50	2.05	7.5	< 0.001	0.78

Covariances

Variables	Estimate	SE	*z*	*P* (bootstr.)	Stand. Est.
**Vertical structural stratification ~~**					
Horizontal structural variation	0.00	0.00	-5.2	< 0.001	-0.51
Trait composition 1	0.05	0.01	4.8	< 0.001	0.43
**Horizontal structural variation ~~**					
Trait composition 1	-0.02	0.01	-3.9	< 0.001	-0.44
**Plant biomass ~~**					
Trait composition 2	-0.11	0.04	-2.6	0.009	-0.35

R^2

Variable	Estimate
Functional diversity	0.47
Trait composition 2	0.04

Plant biomass	0.05
Vertical structural stratification	0.22
Horizontal structural variation	0.16
Arthropod species richness	0.22

Non-significant (P > 0.05 based on 1,000 bootstrap draws) predictors are printed in italics. RMSEA = Root mean square error of approximation.

Supplementary Table 22. Alternative path model for biodiversity effects on *herbivore* species richness in the Jena *grassland experiment, with effects of arthropod abundance factored out by rarefaction*.

**Model**					
AICc initial model	555.8				
AICc minimal model	541.7				
Number of observations	92				
Chi-square	19.1				
P (Chi-square)	0.211				
P (Bollen-Stine Bootstrap)	0.252				
Degrees of freedom	15				
RMSEA	0.054				
95% CI (RMSEA)	0 - 0.119				
P (RMSEA)	0.418				
**Regressions**					
Respone ~ Predictor	Estimate	SE	*z*	*P* (bootstr.)	Stand. Est.
**Functional diversity ~**					
Plant species richness	0.86	0.07	11.5	< 0.001	0.69
**Trait composition 2 ~**					
Plant species richness	*-0.46*	*0.26*	*-1.8*	*0.076*	*-0.19*
**Plant biomass ~**					
Plant species richness	0.14	0.07	2.0	0.040	0.23
**Vertical structural stratification ~**					
Plant species richness	0.03	0.01	2.6	0.008	0.21
Plant biomass	0.09	0.02	4.5	< 0.001	0.38
**Horizontal structural variation ~**					
Plant biomass	-0.04	0.01	-4.3	< 0.001	-0.40
**Arthropod species richness ~**					
Trait composition 1	1.61	0.76	2.1	0.035	0.22
Trait composition 2 ~	-0.19	0.07	-2.8	0.005	-0.24
Vertical structural stratification	10.55	1.78	5.9	< 0.001	0.57
**Variances**					
Variable	Estimate	SE	*z*	*P* (bootstr.)	Stand. Est.
Plant species richness	0.24	0.03	7.5	< 0.001	1.00
Functional diversity	0.20	0.03	6.9	< 0.001	0.53
Trait composition 1	2.76	0.39	7.0	< 0.001	1.00
Trait composition 2	1.34	0.26	5.2	< 0.001	0.96
Plant biomass	0.08	0.01	6.9	< 0.001	0.95
Vertical structural stratification	0.00	0.00	8.6	< 0.001	0.78
Horizontal structural variation	0.00	0.00	7.0	< 0.001	0.84
Arthropod species richness	1.20	0.14	8.5	< 0.001	0.67
**Covariances**					
Variables	Estimate	SE	*z*	*P* (bootstr.)	Stand. Est.
**Vertical structural stratification ~~**					
Horizontal structural variation	0.00	0.00	-5.0	< 0.001	-0.51
Trait composition 1	0.05	0.01	4.7	< 0.001	0.43
**Horizontal structural variation ~~**					
Trait composition 1	-0.02	0.01	-3.9	< 0.001	-0.44
**Plant biomass ~~**					
Trait composition 2	-0.11	0.04	-2.6	0.008	-0.35
**R^2**					
Variable	Estimate				
Functional diversity	0.47				
Trait composition 2	0.04				
Plant biomass	0.05				

Vertical structural stratification	0.22
Horizontal structural variation	0.16
Arthropod species richness	0.33

Non-significant (*P* > 0.05 based on 1,000 bootstrap draws) predictors are printed in italics. RMSEA = Root mean square error of approximation.

Supplementary Table 23. Alternative path model for biodiversity effects on *predator* species richness in the Jena *grassland experiment, with effects of arthropod abundance factored out by rarefaction*.

**Model**	
AICc initial model	706.8
AICc minimal model	690.4
Number of observations	92
Chi-square	23.5
P (Chi-square)	0.134
P (Bollen-Stine Bootstrap)	0.163
Degrees of freedom	17
RMSEA	0.064
95% CI (RMSEA)	0 - 0.122
P (RMSEA)	0.323

Regressions

Respone ~ Predictor	Estimate	SE	*z*	*P* (bootstr.)	Stand. Est.
**Functional diversity ~**					
Plant species richness	0.86	0.07	12.0	< 0.001	0.69
**Trait composition 2 ~**					
Plant species richness	*-0.46*	*0.26*	*-1.8*	*0.075*	*-0.19*
**Plant biomass ~**					
Plant species richness	0.14	0.07	2.0	0.043	0.23
**Vertical structural stratification ~**					
Plant species richness	0.03	0.01	2.6	0.008	0.21
Plant biomass	0.09	0.02	4.7	< 0.001	0.38
**Horizontal structural variation ~**					
Plant biomass	-0.04	0.01	-4.5	< 0.001	-0.40
**Arthropod species richness ~**					
Plant biomass	3.88	0.86	4.5	< 0.001	0.40

Variances

Variable	Estimate	SE	*z*	*P* (bootstr.)	Stand. Est.
Plant species richness	0.24	0.03	7.0	< 0.001	1.00
Functional diversity	0.20	0.03	7.6	< 0.001	0.53
Trait composition 1	2.76	0.40	6.8	< 0.001	1.00
Trait composition 2	1.34	0.25	5.5	< 0.001	0.96
Plant biomass	0.08	0.01	7.0	< 0.001	0.95
Vertical structural stratification	0.00	0.00	8.4	< 0.001	0.78
Horizontal structural variation	0.00	0.00	7.2	< 0.001	0.84
Arthropod species richness	6.52	0.91	7.2	< 0.001	0.84

Covariances

Variables	Estimate	SE	*z*	*P* (bootstr.)	Stand. Est.
**Vertical structural stratification ~~**					
Horizontal structural variation	0.00	0.00	-5.1	< 0.001	-0.51
Trait composition 1	0.05	0.01	4.6	< 0.001	0.43
**Horizontal structural variation ~~**					
Trait composition 1	-0.02	0.01	-3.9	< 0.001	-0.44
**Plant biomass ~~**					
Trait composition 2	-0.11	0.04	-2.6	0.009	-0.35

R²

Variable	Estimate
Functional diversity	0.47
Trait composition 2	0.04
Plant biomass	0.05
Vertical structural stratification	0.22
Horizontal structural variation	0.16

Arthropod species richness	0.16

Non-significant (*P* > 0.05 based on 1,000 bootstrap draws) predictors are printed in italics. RMSEA = Root mean square error of approximation.

Supplementary Table 24 Alternative path model for biodiversity effects on *parasitoid* species richness in the Jena *grassland experiment, with effects of arthropod abundance factored out by rarefaction*.

**Model**	
AICc initial model	152.8
AICc minimal model	133.3
Number of observations	92
Chi-square	23.6
P (Chi-square)	0.169
P (Bollen-Stine Bootstrap)	0.320
Degrees of freedom	18
RMSEA	0.058
95% CI (RMSEA)	0 - 0.116
P (RMSEA)	0.382

Regressions

Respone ~ Predictor	Estimate	SE	*z*	*P* (bootstr.)	Stand. Est.
**Functional diversity ~**					
Plant species richness	0.86	0.08	11.0	< 0.001	0.69
**Trait composition 2 ~**					
Plant species richness	*-0.46*	*0.27*	*-1.7*	*0.088*	*-0.19*
**Plant biomass ~**					
Plant species richness	0.14	0.07	2.0	0.043	0.23
**Vertical structural stratification ~**					
Plant species richness	0.03	0.01	2.6	0.010	0.21
Plant biomass	0.09	0.02	4.7	< 0.001	0.38
**Horizontal structural variation ~**					
Plant biomass	-0.04	0.01	-4.4	< 0.001	-0.40

Variances

Variable	Estimate	SE	*z*	*P* (bootstr.)	Stand. Est.
Plant species richness	0.24	0.03	7.0	< 0.001	1.00
Functional diversity	0.20	0.03	7.2	< 0.001	0.53
Trait composition 1	2.76	0.40	6.9	< 0.001	1.00
Trait composition 2	1.34	0.25	5.3	< 0.001	0.96
Plant biomass	0.08	0.01	7.0	< 0.001	0.95
Vertical structural stratification	0.00	0.00	9.0	< 0.001	0.78
Horizontal structural variation	0.00	0.00	6.9	< 0.001	0.84
Arthropod species richness	*0.02*	*0.01*	*1.9*	*0.054*	*1.00*

Covariances

Variables	Estimate	SE	*z*	*P* (bootstr.)	Stand. Est.
**Vertical structural stratification ~~**					
Horizontal structural variation	0.00	0.00	-5.2	< 0.001	-0.51
Trait composition 1	0.05	0.01	4.5	< 0.001	0.43
**Horizontal structural variation ~~**					
Trait composition 1	-0.02	0.01	-3.7	< 0.001	-0.44
**Plant biomass ~~**					
Trait composition 2	-0.11	0.04	-2.7	0.008	-0.35

R^2

Variable	Estimate
Functional diversity	0.47
Trait composition 2	0.04
Plant biomass	0.05
Vertical structural stratification	0.22
Horizontal structural variation	0.16

Non-significant ($P > 0.05$ based on 1,000 bootstrap draws) predictors are printed in italics. RMSEA = Root mean square error of approximation.

Supplementary Table 25. Dimension reduction of community-weighted mean trait values (CWMs) of the BEF-China forest experiment.

	PC1	PC2
Specific leaf area	**-0.58**	0.11
Leaf dry matter content	0.37	**0.54**
Leaf nitrogen concentration	**-0.54**	-0.06
Leaf carbon concentration	0.13	**-0.83**
Leaf toughness	**0.47**	-0.13
% explained	0.43	0.23
Cumulative % explained	0.43	0.66

Loadings and eigenvalues of principal components (PC) selected from a principal components analysis (PCA) on the CWM of leaf traits (most influential variables in bold).

Supplementary Table 26. Dimension reduction of community-weighted mean trait values (CWMs) of the Jena experiment.

	PC1	PC2
Leaf dry matter content	**0.50**	-0.28
Specific leaf area	0.01	**-0.54**
Leaf nitrogen concentration	**-0.47**	-0.32
Leaf carbon concentration	-0.14	**-0.71**
Leaf silica concentration	**0.54**	-0.08
Leaf toughness	**0.47**	-0.14
% explained	0.46	0.24
Cumulative % explained	0.46	0.70

Loadings and eigenvalues of principal components (PC) selected from a principal components analysis (PCA) on the CWM of leaf traits (most influential variables in bold).

Supplementary Table 27. Dimension reduction of predictors related to vertical stratification (Rao's Q) and horizontal variability (Moran's I; increasing values indicate an increase in spatial aggregation of structurally similar trees, i.e. decreasing random patterning of structural diversity.

	PC1	PC2
Rao's Q tree height	**0.64**	0.29
Rao's Q crown projection area	**0.60**	0.39
Moran's I tree height	0.31	**-0.65**
Moran's I crown projection area	0.37	**-0.59**
% explained	0.44	0.37
Cumulative % explained	0.44	0.81

Negative values indicate higher spatial variability across trees of the study plots) of the BEF-China forest experiment. Loadings and eigenvalues of principal components (PC) selected from a principal components analysis (PCA; most influential variables in bold). Values for PC1 were multiplied by -1 to represent increasing diversity.

Supplementary References

1 Durka, W. & Michalski, S. G. Daphne: a dated phylogeny of a large European flora for phylogenetically informed ecological analyses. *Ecology* **93**, 2297-2297 (2012).

2 Purschke, O., Michalski, S. G., Bruelheide, H. & Durka, W. Phylogenetic turnover during subtropical forest succession across environmental and phylogenetic scales. *Ecol. Evol.* **7**, 11079-11091 (2017).
