# Supplementary material for: Multiple plant diversity components drive consumer communities across ecosystems
Source: Nat Commun. 2019 Mar 29;10:1460. doi: 10.1038/s41467-019-09448-8 (PMC6440984; doi:10.1038/s41467-019-09448-8)
Supplement: Supplementary file 3 — Reporting Summary [file 41467_2019_9448_MOESM3_ESM.pdf]

nature research

Corresponding author(s): Schuldt

Last updated by author(s): Feb 25, 2019

Reporting Summary

Nature Research wishes to improve the reproducibility of the work that we publish. This form provides structure for consistency and transparency in reporting. For further information on Nature Research policies, see Authors & Referees and the Editorial Policy Checklist.

Statistics

For all statistical analyses, confirm that the following items are present in the figure legend, table legend, main text, or Methods section.

n/a	Confirmed	
☐	☒	The exact sample size (*n*) for each experimental group/condition, given as a discrete number and unit of measurement
☐	☒	A statement on whether measurements were taken from distinct samples or whether the same sample was measured repeatedly
☐	☒	The statistical test(s) used AND whether they are one- or two-sided<br>*Only common tests should be described solely by name; describe more complex techniques in the Methods section.*
☐	☒	A description of all covariates tested
☐	☒	A description of any assumptions or corrections, such as tests of normality and adjustment for multiple comparisons
☐	☒	A full description of the statistical parameters including central tendency (e.g. means) or other basic estimates (e.g. regression coefficient) AND variation (e.g. standard deviation) or associated estimates of uncertainty (e.g. confidence intervals)
☐	☒	For null hypothesis testing, the test statistic (e.g. *F*, *t*, *r*) with confidence intervals, effect sizes, degrees of freedom and *P* value noted<br>*Give P values as exact values whenever suitable.*
☒	☐	For Bayesian analysis, information on the choice of priors and Markov chain Monte Carlo settings
☒	☐	For hierarchical and complex designs, identification of the appropriate level for tests and full reporting of outcomes
☐	☒	Estimates of effect sizes (e.g. Cohen's *d*, Pearson's *r*), indicating how they were calculated

Our web collection on statistics for biologists contains articles on many of the points above.

Software and code

Policy information about availability of computer code

Data collection	No software used
Data analysis	All analyses were conducted in R 3.3.1 (www.r-project.org) with the packages 'vegan', 'FD', 'VoxR', and 'lavaan'.

For manuscripts utilizing custom algorithms or software that are central to the research but not yet described in published literature, software must be made available to editors/reviewers. We strongly encourage code deposition in a community repository (e.g. GitHub). See the Nature Research guidelines for submitting code & software for further information.

Data

Policy information about availability of data

All manuscripts must include a data availability statement. This statement should provide the following information, where applicable:

- Accession codes, unique identifiers, or web links for publicly available datasets
- A list of figures that have associated raw data
- A description of any restrictions on data availability

Data availability: Data used in the analyses will be available on the data repository of the German Centre of Integrative Biodiversity Research (iDiv) at https://idata.idiv.de/.
A reporting summary for this Article is available as a Supplementary Information file. The source data underlying Figs 1-3 and Supplementary Fig. 2 are provided as a Source Data file.

Field-specific reporting

Please select the one below that is the best fit for your research. If you are not sure, read the appropriate sections before making your selection.

☐ Life sciences ☐ Behavioural & social sciences ☒ Ecological, evolutionary & environmental sciences

For a reference copy of the document with all sections, see nature.com/documents/nr-reporting-summary-flat.pdf

Ecological, evolutionary & environmental sciences study design

All studies must disclose on these points even when the disclosure is negative.

Study description	Analysis of two large-scale biodiversity experiments (Jena Experiment in grasslands: N = 92 study plots, BEF-China experiment in forests: N = 46 study plots). Path models to explain plot-level abundance and species richness of arthropods with variables related to multiple components of plant diversity (plant species richness, functional diversity, trait composition, biomass, structural diversity). Both experiments manipulated plant species richness (grassland: 1-8 species, forest: 1-24 species) with replicated sets of species mixtures.
Research sample	All arthropods sampled in the two experiments.
Sampling strategy	Arthropods were sampled in both experiments in 2014, using quantitative methods best suited for a representative assessment of their diversity in each ecosystem: branch beating, standardized assessments of trophobioses (mutualistic interactions between ants and hemipterans), and trap nests in the forest system; pitfall traps and suction sampling in the grassland system. Sample size was based on established knowledge regarding the specific methods and followed published studies (e.g. Scherber et al. 2010 Nature in the grassland system) or made sure that spatial variation of plot conditions was taken into account (e.g. sampling 40 individual trees by beating in the forest system to make sure that all tree species per plot are included with replicates in the sampling).
Data collection	All data were collected by the authors of the manuscript. All arthropod data were collected in 2014, using the set of sampling methods described above. Data on plant structural diversity were collected in 2014, using terrestrial laser scanning (grassland) or direct measurements in the field (measuring tapes, forest system). Plant biomass was determined in 2014 based on harvested plant material (grassland) or estimates using measurements of tree height and basal area (forest). Leaf traits were measured on plants of the study plots between 2005 and 2012.
Timing and spatial scale	Arthropod data were assessed at the level of individual study plots during the main growing season in each ecosystem in 2014. Depending on the sampling method, sampling was continuous over the entire main growing season (pitfall traps in grassland, trap-nests in forest) or used representative points in time (twice per year in early and late season: suction sampling in grassland, beating in forest) with peak arthropod activity. Data on plant structural diversity and biomass were assessed before mowing events in grassland (twice per year) and at the end of the main growing season in forest (september 2014).
Data exclusions	No data were excluded in the grassland system (i.e. all study plots for which data on plant structure was available were used). In the forest system, several study plots had to be excluded because of lack of or limited tree establishment (8 plots) and lack of arthropod sampling (10 plots without trap-nest sampling).
Reproducibility	Reproducibility of the experiment (which manipulated plant species richness) was ensured by having multiple replicates of each level of plant species richness that differed in plant species composition and plant species pool.
Randomization	The treatment variable of both experiments was plant species richness. Study plots with different levels of plant species richness were randomly distributed across the study sites. In the grassland system, plants established from sown seed mixtures. In the forest system, tree species were randomly assigned to individual planting positions within the plots, with the total number of individuals per plot divided equally among the species planted in a given plot.
Blinding	Blinding was not relevant because sampling followed the above described randomized study design and because no selection of arthropod groups was done in the field (i.e. all arthropods were sampled).

Did the study involve field work? ☒ Yes ☐ No

Field work, collection and transport

Field conditions	BEF-China forest site: The mean annual temperature at the study site is 16.7°C, and mean annual precipitation is 1,800 mm. Jena Experiment grassland site: The mean annual temperature at the study site is 9.9°C, and mean annual precipitation is 610 mm. In case of individual sampling events, sampling was conducted under dry and warm weather conditions.
Location	The BEF-China forest experiment is located close to Xingangshan, Jianxi Province, China (29°08′–29°11′ N, 117°90′–117°93′ E, 100 – 300 m above sea level) and represents subtropical mixed evergreen broadleaved forest. The Trait-Based Experiment, one of the experimental grassland experiments running in the framework of the Jena Experiment, is located close to Jena, Thuringia, Germany (50°55′N, 11°35′E; 130 m above sea level) and represents mesophilic temperate grasslands.
Access and import/export	Both study sites were rented for the duration of the experiments. Our study conforms to the legal requirements of the People's Republic of China. Access to the experiment is granted through the land-renting contract with the Xingangshan Forestry Co. Ltd.,

	Dexing, Jiangxi Province, China (04.11.2008). Sharing samples and data is provided through an agreement with the Institute of Botany, Chinese Academy of Sciences, Beijing, 10093 (05.2010). Arthropod data were processed in the respective countries.
Disturbance	Sampling of arthropods was either passive (pitfall traps, trap-nests) or involved non-destructive active sampling methods (suction sampling, beating).

Reporting for specific materials, systems and methods

We require information from authors about some types of materials, experimental systems and methods used in many studies. Here, indicate whether each material, system or method listed is relevant to your study. If you are not sure if a list item applies to your research, read the appropriate section before selecting a response.

Materials & experimental systems

n/a	Involved in the study	
☒	☐	Antibodies
☒	☐	Eukaryotic cell lines
☒	☐	Palaeontology
☒	☐	Animals and other organisms
☒	☐	Human research participants
☒	☐	Clinical data

Methods

n/a	Involved in the study	
☒	☐	ChIP-seq
☒	☐	Flow cytometry
☒	☐	MRI-based neuroimaging
