# Supplementary material for: Multiple plant diversity components drive consumer communities across ecosystems
Source: Nat Commun. 2019 Mar 29;10:1460. doi: 10.1038/s41467-019-09448-8 (PMC6440984; doi:10.1038/s41467-019-09448-8)
Supplement: Supplementary file 4 — Description of Additional Supplementary Files [file 41467_2019_9448_MOESM4_ESM.pdf]

Description of Additional Supplementary Files

File Name: Supplementary Data 1
Description: List of arthropod species and trophic level for the grassland and forest experiments
